# Supplementary material for: Calcium-mediated rapid movements defend against herbivorous insects in Mimosa pudica
Source: Nat Commun. 2022 Nov 14;13:6412. doi: 10.1038/s41467-022-34106-x (PMC9663552; doi:10.1038/s41467-022-34106-x)
Supplement: Supplementary file 1 — Supplementary Information [file 41467_2022_34106_MOESM1_ESM.pdf]

Calcium-mediated rapid movements defend against herbivorous insects in *Mimosa pudica*

Hagihara et al.

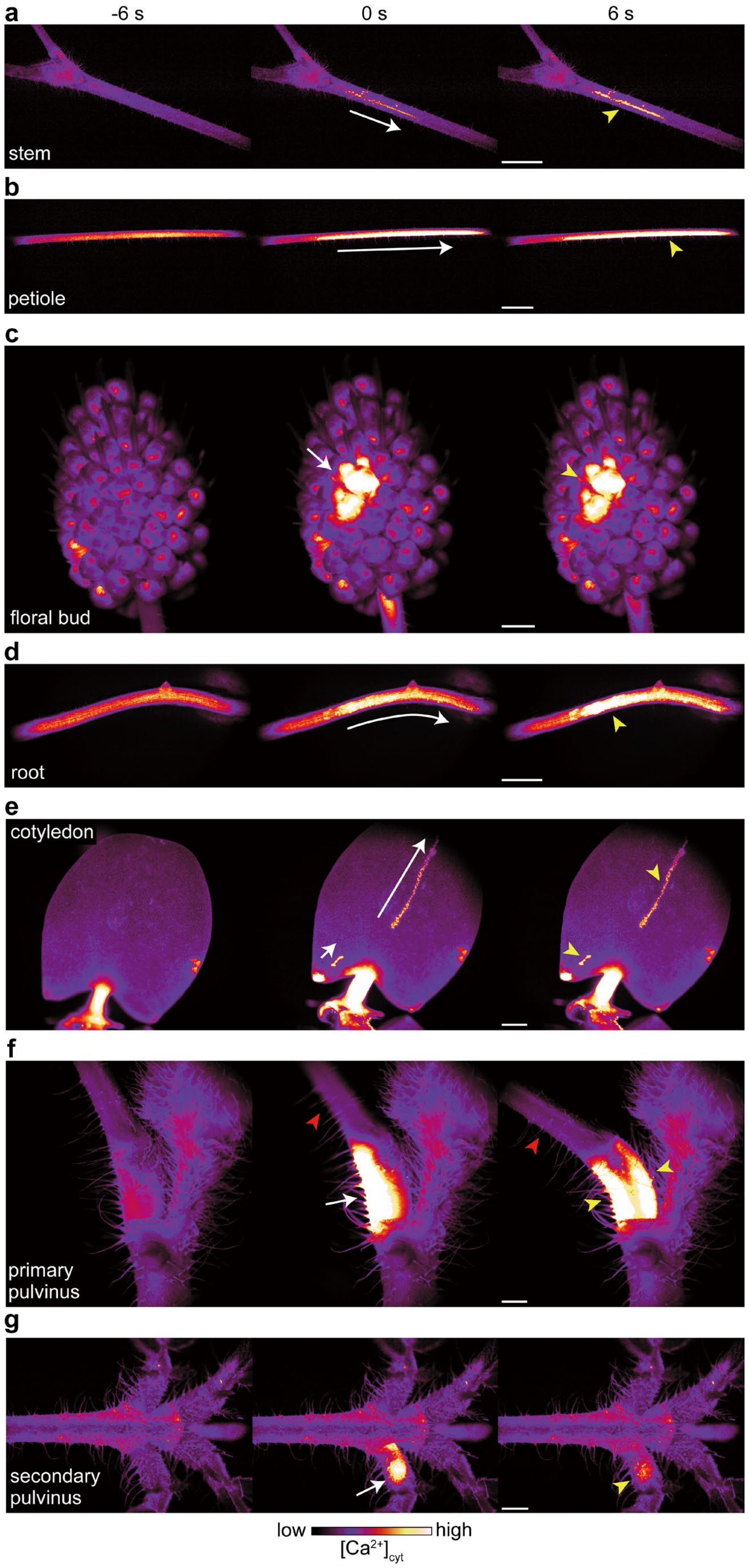

a
-6 s
0 s
6 s
stem
b
petiole
c
floral bud
d
root
e
cotyledon
f
primary
pulvinus
g
secondary
pulvinus
low
high
[Ca2+]cyt

Supplementary Fig. 1 | GCaMP6f fluorescence in various tissues in established transgenic *M. pudica*. a–**g** Touch by tweezers or a micropipette tip (white arrow, 0 s) caused $[Ca^{2+}]_{cyt}$ increases (yellow arrowheads) in the stem (**a**), petiole (**b**), floral bud (**c**), root (**d**), cotyledon (**e**), primary pulvinus (**f**, stipules were removed), and secondary pulvinus (**g**). For $[Ca^{2+}]_{cyt}$ changes in a tertiary pulvinus, leaflet, and rachilla, see Figs. 1a–c and 2a. In **f**, petiole displacement is denoted by red arrowheads. Scale bars, 5 mm (**a**–**c**) or 1 mm (**d**–**g**).

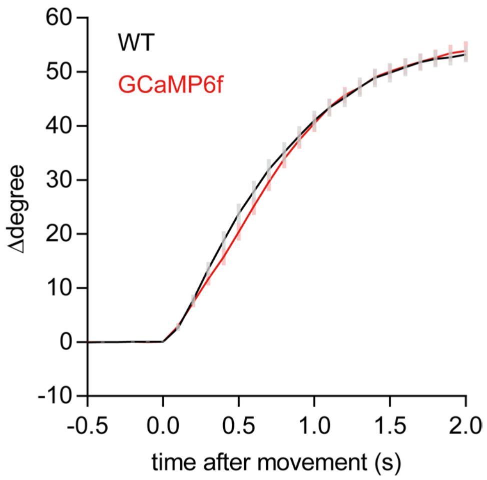

Supplementary Fig. 2 | Ectopic GCaMP6f expression does not greatly affect rapid movement. Leaflet angle changes were triggered by wounding leaflets and monitored over time in the WT (black, n = 10) and GCaMP6f leaves (red, n = 10). Mean ± SEM values are shown. The third leaf from the top of the stem was cut from 2-month-old plants and used for this experiment.

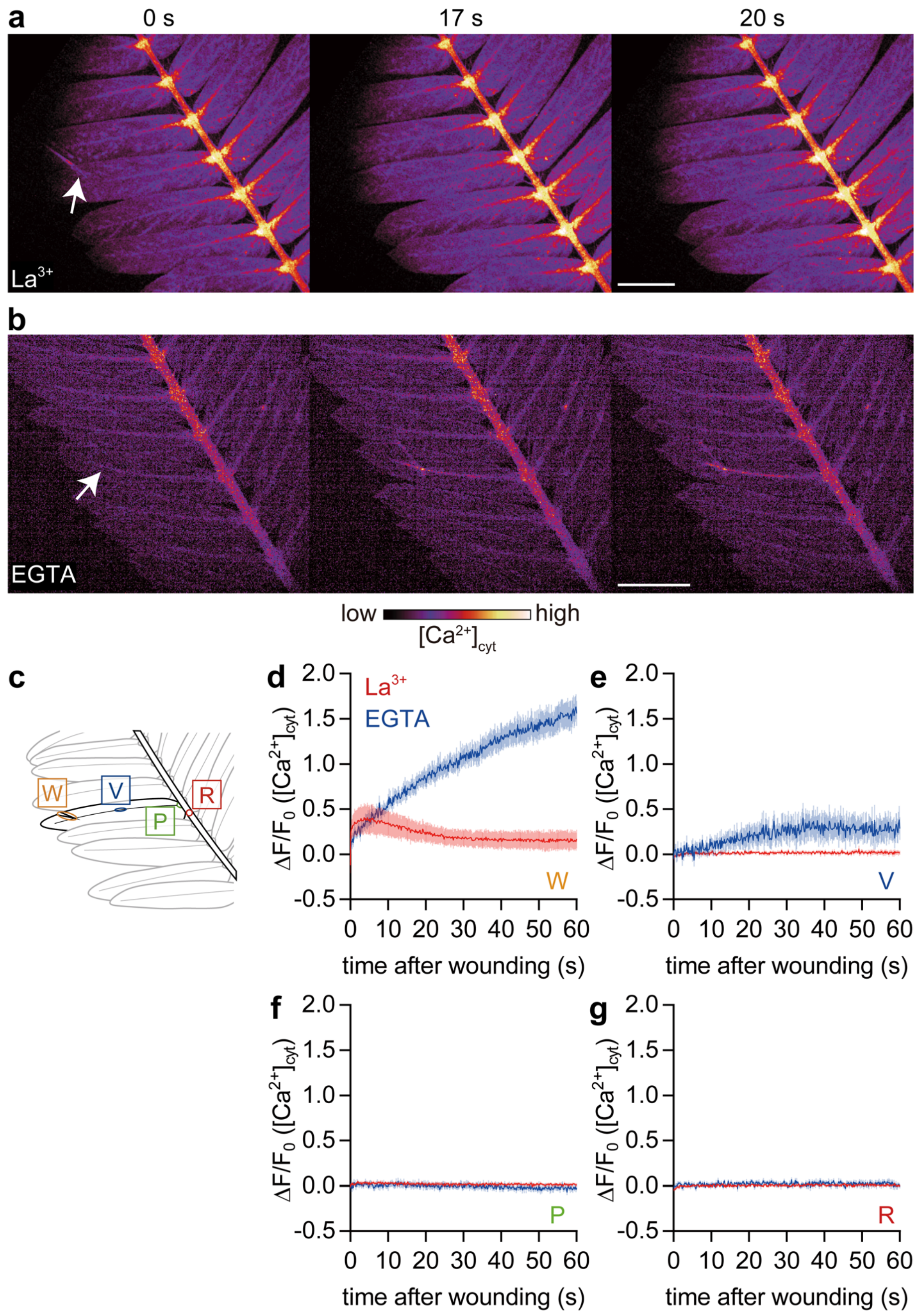

Supplementary Fig. 3 | La^{3+} and EGTA pretreatments block long-range $[Ca^{2+}]_{cyt}$ signals. a, **b** 50 mM La^{3+} (**a**) and 50 mM EGTA (**b**) pretreatments retarded the long-distance propagation of the $[Ca^{2+}]_{cyt}$ increase in leaves upon wounding (white arrows, 0 s). **c** Diagram of the leaf with the ROIs for $[Ca^{2+}]_{cyt}$ analysis. W, wound site; V, leaflet vein; P, tertiary pulvinus; R, rachilla. **d–g** Wound-induced $[Ca^{2+}]_{cyt}$ changes at the wound site (**d**), leaflet vein (**e**), tertiary pulvinus (**f**), and rachilla (**g**) in leaves treated with 50 mM La^{3+} and 50 mM EGTA (*n* = 7 each). Mean ± SEM values are shown. Scale bars, 5 mm.

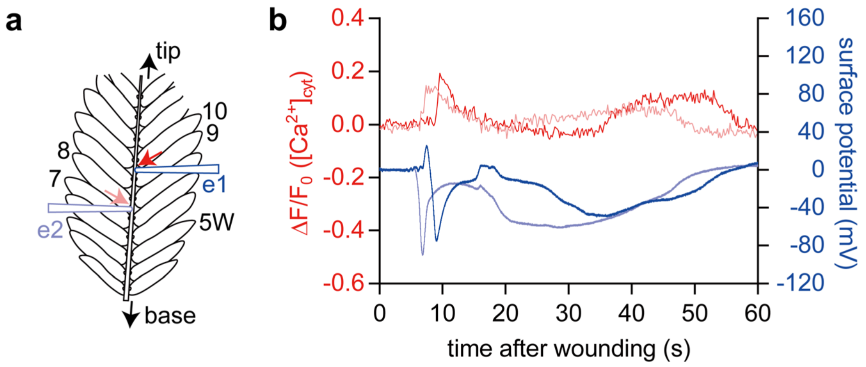

Supplementary Fig. 4 | Acropetal propagation of $[Ca^{2+}]_{cyt}$ and electrical signals. a Electrodes (e1 and e2, blue rectangles) and ROIs (red arrows, 1 mm from the electrodes) were set on a rachilla for surface potential measurements and $[Ca^{2+}]_{cyt}$ analysis, respectively. A pair of leaflets was numbered from the base of a pinna. W, wounding. **b** Changes in $[Ca^{2+}]_{cyt}$ and surface potential in response to wounding (colors as depicted in **a**).

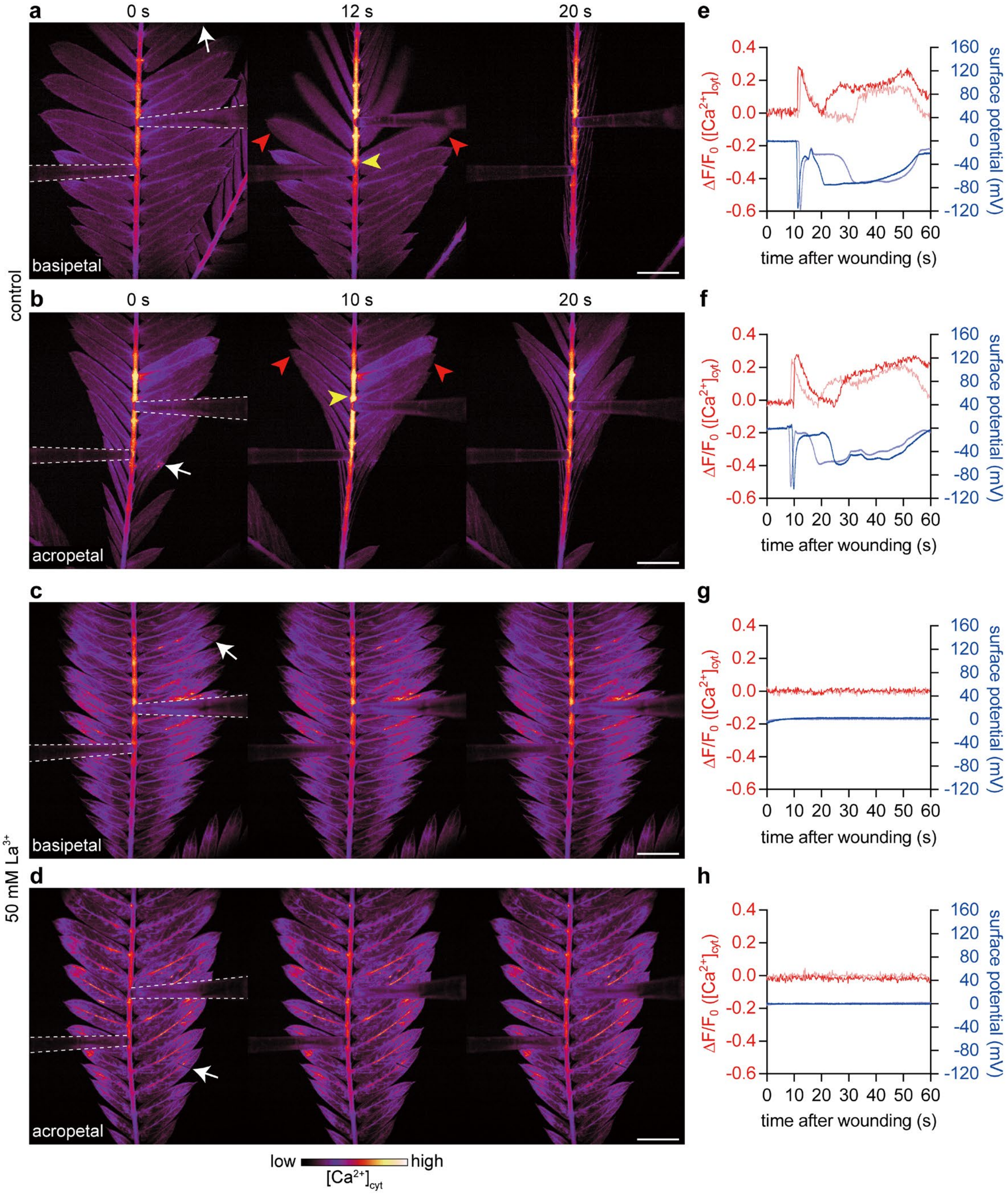

Supplementary Fig. 5 | La^{3+} inhibits the transmission of both $[Ca^{2+}]_{cyt}$ and electrical signals. a–**d** Wounding (white arrows, 0 s) induced $[Ca^{2+}]_{cyt}$ signals (yellow arrowhead) that basipetally (**a**) and acropetally (**b**) propagated through the rachilla and leaflet movements (red arrowheads) in the control leaves but not in the leaves treated with 50 mM La^{3+} (**c** and **d**). **e**–**h** $[Ca^{2+}]_{cyt}$ and electrical signals in control leaves (basipetal propagation, **e**; acropetal propagation, **f**) and La^{3+}-treated leaves (basipetal propagation, **g**; acropetal propagation, **h**) upon wounding. Typical data are displayed (*n*

= 5 for **a**, **b**, **e**, and **f**; $n = 6$ for **d** and **h**; and $n = 8$ for **c** and **g**). Dashed outlines in the snapshots highlight the electrodes set on the rachillae. Scale bars, 5 mm.

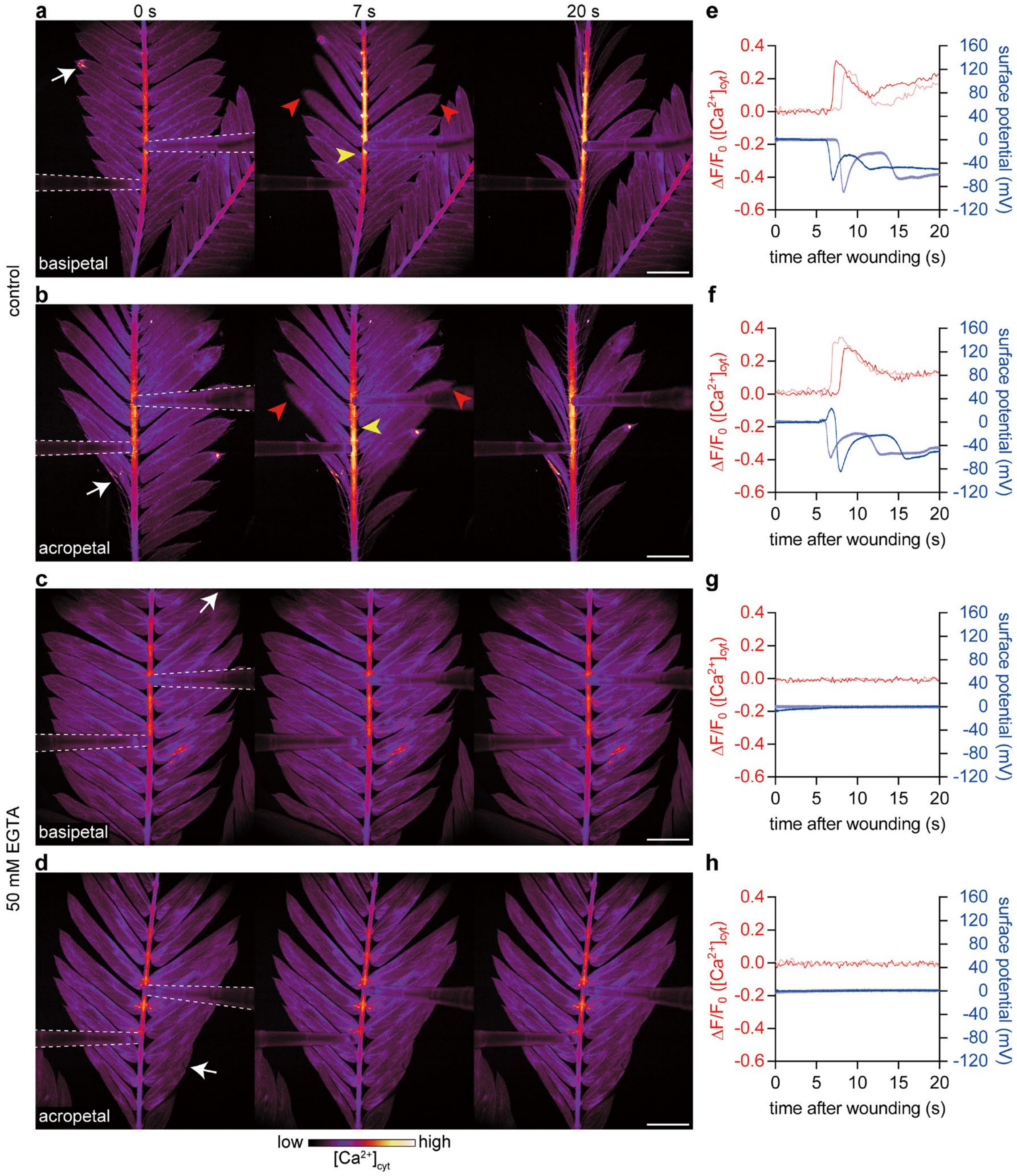

Supplementary Fig. 6 | EGTA inhibits both $[Ca^{2+}]_{cyt}$ and electrical signals. a–d Wounding (white arrows, 0 s) caused basipetal (**a**) and acropetal (**b**) propagations of the $[Ca^{2+}]_{cyt}$ increases (yellow arrowheads) and leaflet movements (red arrowheads) in control leaves but not in EGTA-treated leaves (50 mM; **c** and **d**). **e–h** $[Ca^{2+}]_{cyt}$ signatures and surface potential changes in control leaves (basipetal propagation, **e**; acropetal propagation, **f**) and EGTA-treated leaves (basipetal propagation, **g**; acropetal propagation, **h**) upon wounding. Typical data are displayed (*n* = 4 for **a** and **e**; *n* = 5 for

b and **f**; and $n = 6$ for **c**, **d**, **g**, and **h**). Dashed outlines in the snapshots highlight the electrodes set on the rachillae. Scale bars, 5 mm.

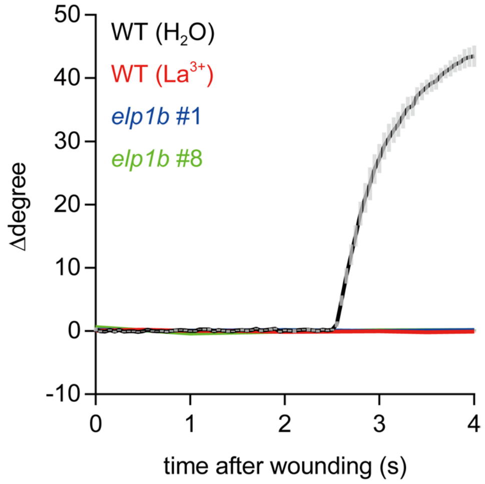

Supplementary Fig. 7 | Leaflet angle changes during wound-induced leaflet movements. Wound-induced leaflet angle changes in the leaves pretreated with H_2O [WT (H_2O), $n = 7$] or 50 mM La^{3+} [WT (La^{3+}), $n = 5$] and the leaves of *elp1b1elp1b2* lines 1 (*elp1b* #1, $n = 5$) and 8 (*elp1b* #8, $n = 5$) were plotted. WT (H_2O) and WT (La^{3+}) data in Fig. 1e and f were reproduced. Mean ± SEM values are shown.

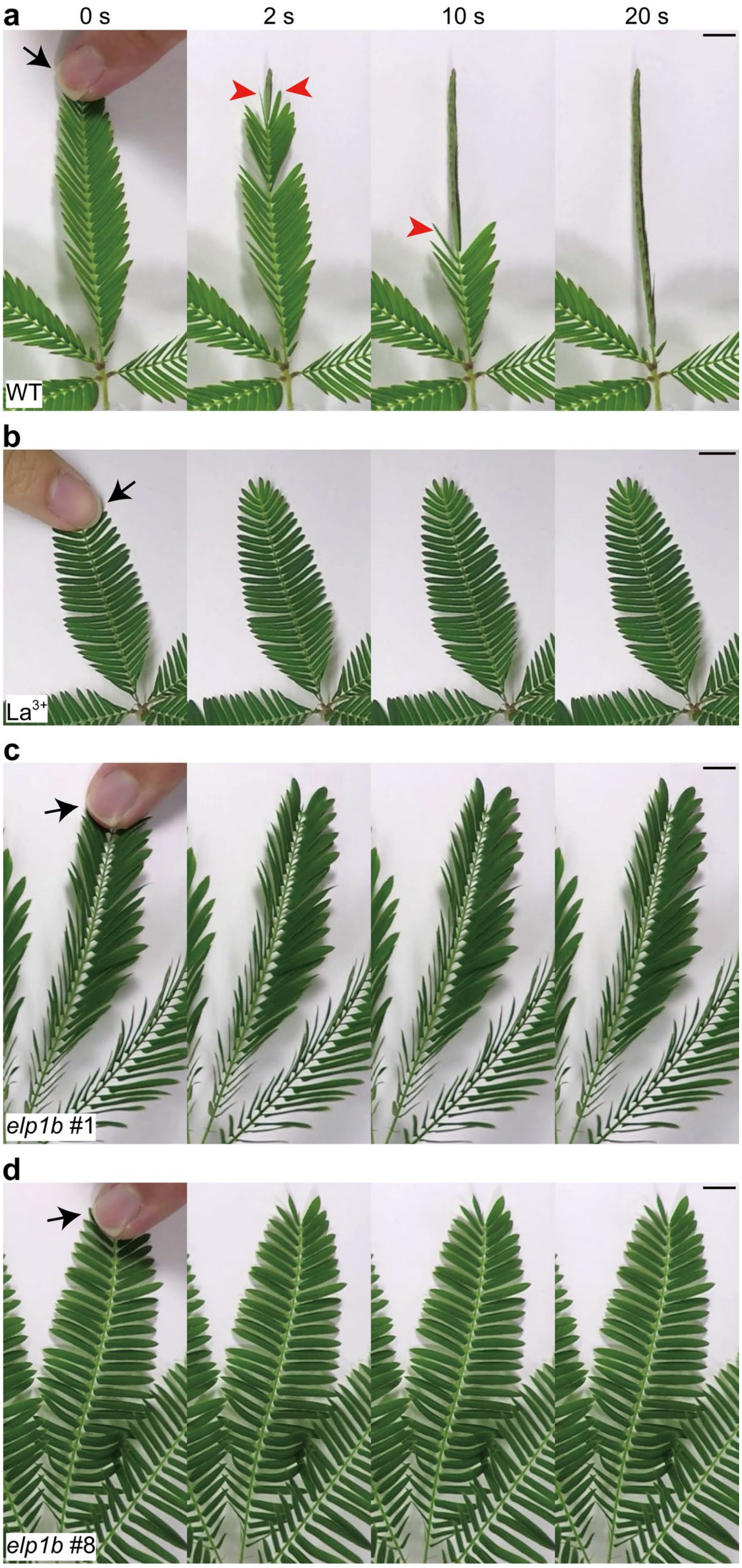

Supplementary Fig. 8 | Leaflet movements in response to touch. a Touch (black arrow, 0 s) caused leaflet movements (red arrowheads) throughout a pinna. **b**–**d** Touch

(black arrows, 0 s) failed to elicit leaflet movements in the leaf pretreated with 50 mM La^{3+} (**b**) and the leaves of *elp1b1elp1b2* lines 1 (*elp1b* #1, **c**) and 8 (*elp1b* #8, **d**). Scale bars, 10 mm.

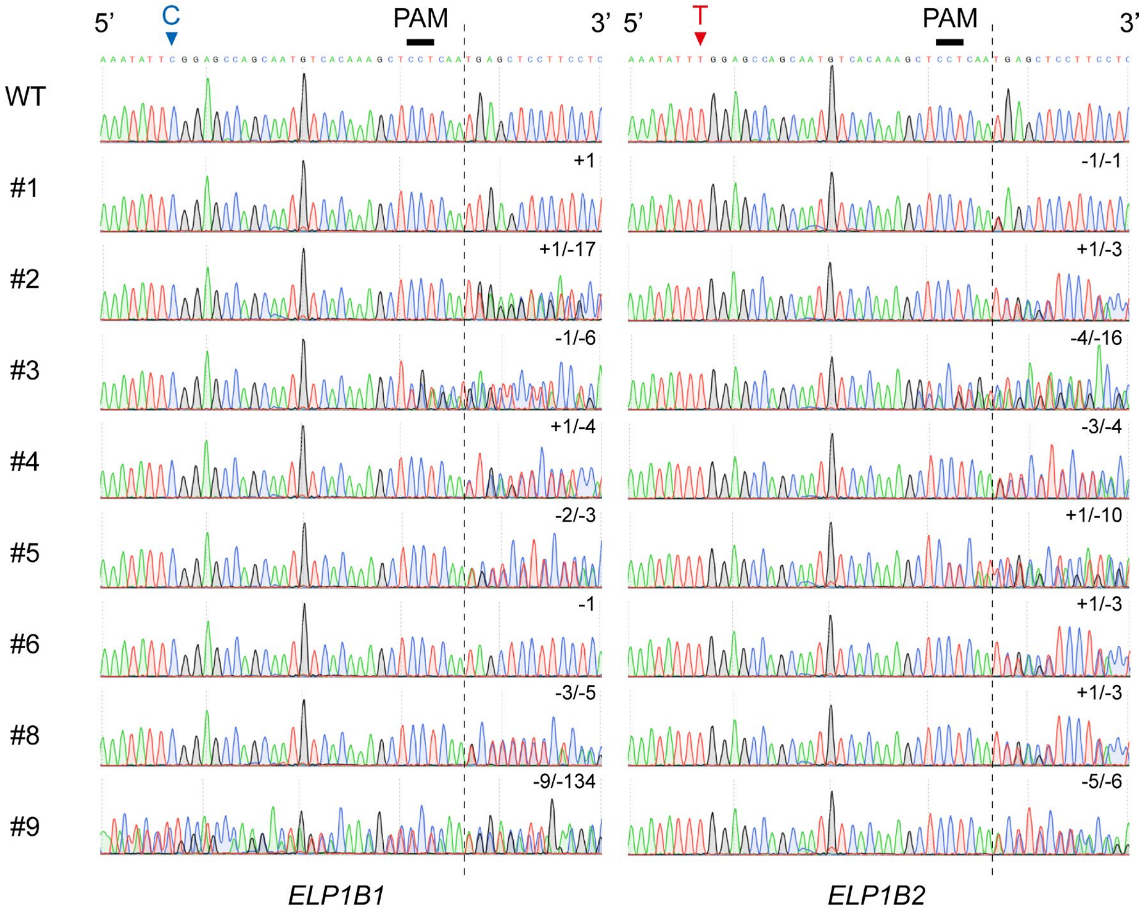

Supplementary Fig. 9 | Sequencing chromatograms of *ELP1B1* and *ELP1B2* genes. Genomic DNA was obtained from wild-type *M. pudica* (WT) or T_0 founders of the CRISPR/Cas9 transgenic lines of 8 independent origins (#1–6 and #8–9). Dashed lines indicate the position of Cas9 cleavage. Arrowheads indicate a discriminative nucleotide between *ELP1B1* and *ELP1B2* genes, which ensured specific PCR amplification of each single gene. Dual peak profiles detected in transgenic founders suggest the presence of two alleles for each gene. Signed numbers in the upper right of each chromatogram indicate the sizes of indels. It could not be distinguished whether the apparent single profile, for example in *ELP1B1* of #1, is ascribed to the same mutation in the two alleles or to a possible failure of PCR amplification caused by a large genomic deletion.

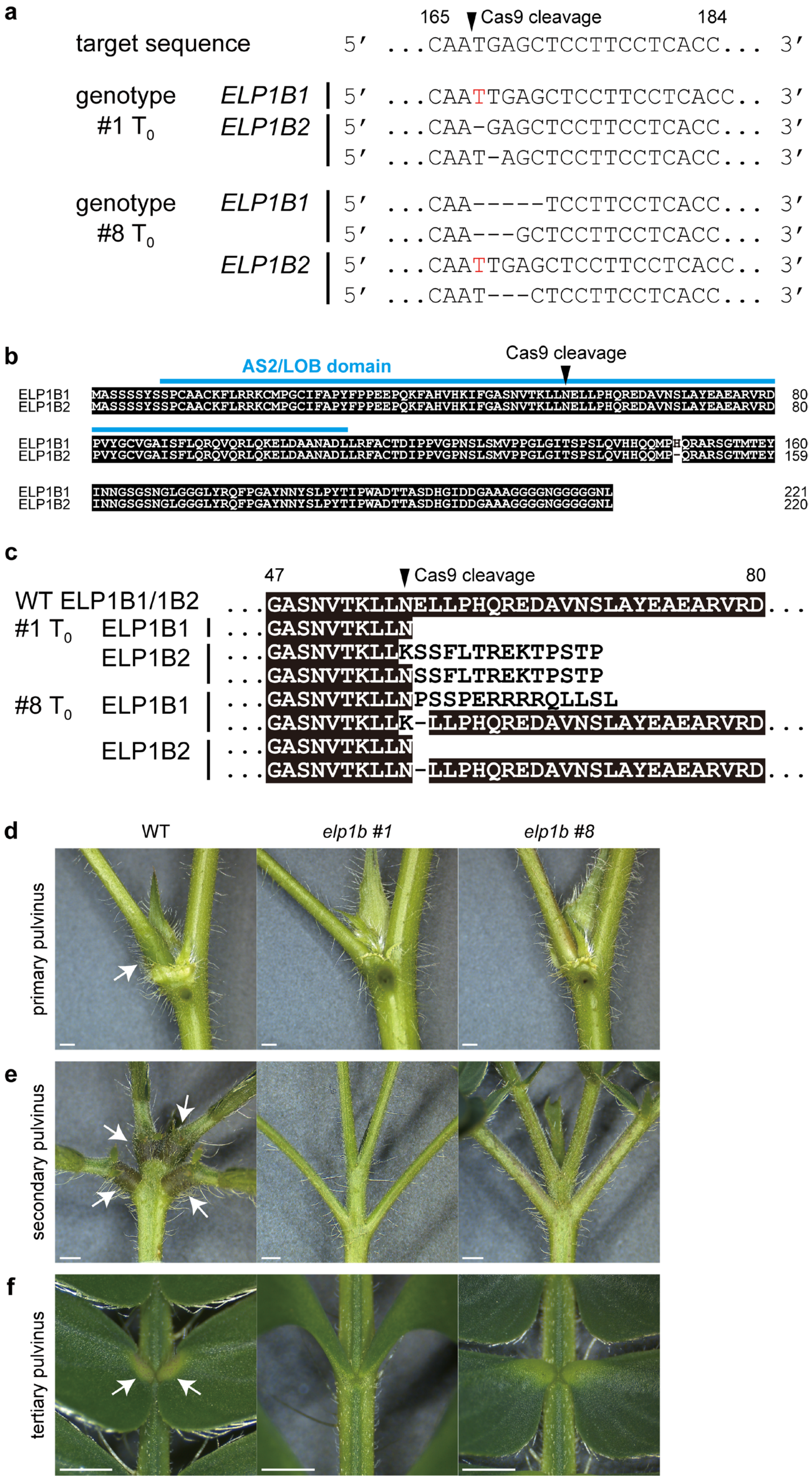
a
165 Cas9 cleavage 184
target sequence 5′ ...CAATGAGCTCCTTCCTCACC... 3′
genotype #1 T_0
ELP1B1 5′ ...CAATTGAGCTCCTTCCTCACC.. 3′
ELP1B2 5′ ...CAA-GAGCTCCTTCCTCACC... 3′
5′ ...CAAT-AGCTCCTTCCTCACC... 3′
genotype #8 T_0
ELP1B1 5′ ...CAA-----TCCTTCCTCACC... 3′
5′ ...CAA---GCTCCTTCCTCACC... 3′
ELP1B2 5′ ...CAATTGAGCTCCTTCCTCACC.. 3′
5′ ...CAAT---CTCCTTCCTCACC... 3′
b
AS2/LOB domain
Cas9 cleavage
ELP1B1 MASSSSYSSPCAACKFLRRKCMPGCIFAPYFPPEEPQKFAHVHKIFGASNVTKLLNELLPHQREDAVNSLAYEAEARVRD 80
ELP1B2 MASSSSYSSPCAACKFLRRKCMPGCIFAPYFPPEEPQKFAHVHKIFGASNVTKLLNELLPHQREDAVNSLAYEAEARVRD 80
ELP1B1 PVYGCVGAISFLQRQVQRLQKELDAANADLLRFACTDIPPVGPNSLSMVPPGLGITSPSLQVHHQQMPHQRARSGTMTEY 160
ELP1B2 PVYGCVGAISFLQRQVQRLQKELDAANADLLRFACTDIPPVGPNSLSMVPPGLGITSPSLQVHHQQMP-QRARSGTMTEY 159
ELP1B1 INNGSGSNGLGGGLYRQFPGAYNNYSLPYTIPWADTTASDHGIDDGAAAGGGGNGGGGGNL 221
ELP1B2 INNGSGSNGLGGGLYRQFPGAYNNYSLPYTIPWADTTASDHGIDDGAAAGGGGNGGGGGNL 220
c
47 Cas9 cleavage 80
WT ELP1B1/1B2 ...GASNVTKLLNELLPHQREDAVNSLAYEAEARVRD...
#1 T_0 ELP1B1 ...GASNVTKLLN
ELP1B2 ...GASNVTKLLKSSFLTREKTPSTP
...GASNVTKLLNSSFLTREKTPSTP
#8 T_0 ELP1B1 ...GASNVTKLLNPSSPERRRRQLLSL
...GASNVTKLLK-LLPHQREDAVNSLAYEAEARVRD...
ELP1B2 ...GASNVTKLLN
...GASNVTKLLN-LLPHQREDAVNSLAYEAEARVRD...
d
WT
elp1b #1
elp1b #8
primary pulvinus
e
secondary pulvinus
f
tertiary pulvinus

Supplementary Fig. 10 | *elp1b1elp1b2* mutants do not develop pulvini. a Genotypes of *ELP1B1* and *ELP1B2* genes in T_0 plants. Red letters and black hyphens denote inserted and deleted nucleotides, respectively. **b** Pairwise alignment of ELP1B1 and ELP1B2 proteins. Amino acid residues conserved between the sequences are highlighted with a black background. **c** Deduced amino acid sequences of remnant ELP1B proteins in *elp1b1elp1b2* double mutants. In T_0 founder #1, all detected mutations were expected to cause frameshift and truncation of the gene products. In T_0 founder #8, 2 of 4 alleles had a 3-bp deletion, which does not cause frameshift and only affects 1 or 2 codons around the mutation. **d–f** The primary (**d**), secondary (**e**), and tertiary pulvini (**f**) of WT did not develop in *elp1b1elp1b2* mutants. In **d**, one of the stipules at the leaf bases was removed. Note that the *elp1b1elp1b2* line 8 (*elp1b* #8) leaf has tertiary pulvinus-like structures. Scale bars, 1 mm.

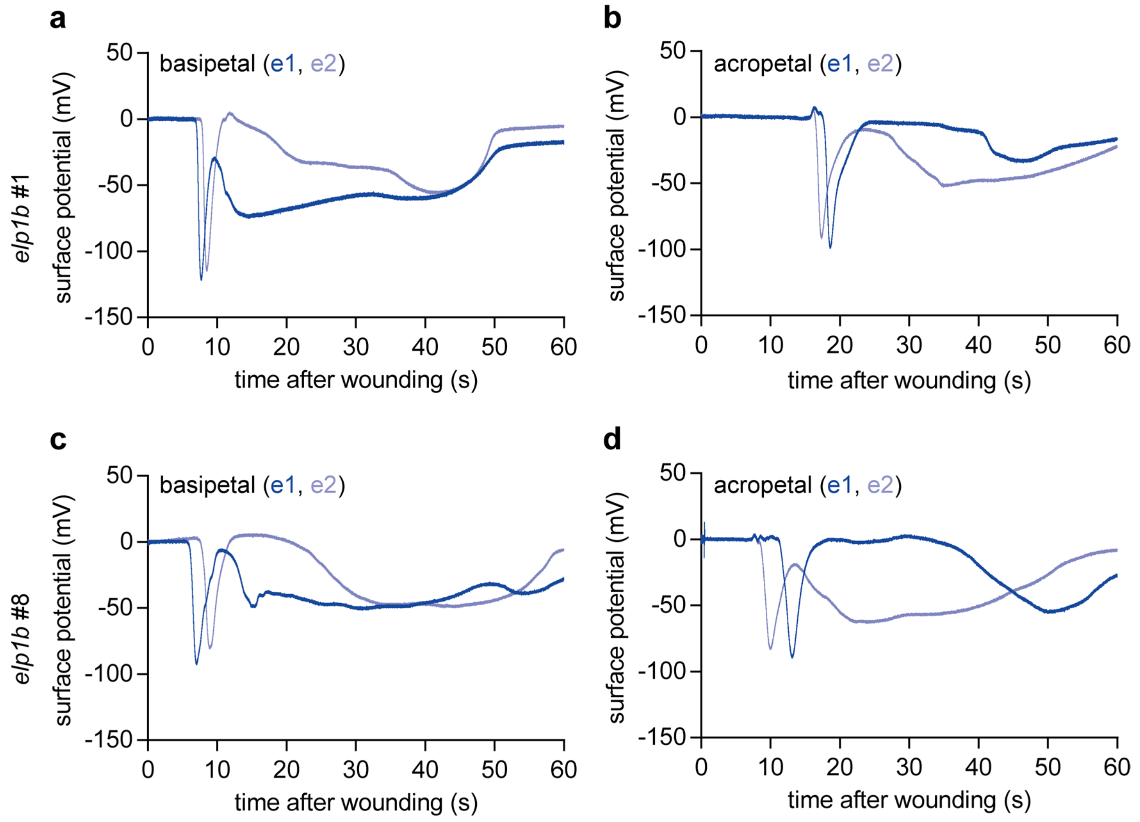

Supplementary Fig. 11 | Wound-induced electrical signals in *elp1b1elp1b2* mutant leaves. **a**, **b** Upon wounding a leaflet, electrical signals were propagated through the rachillae basipetally (**a**) and acropetally (**b**) in *elp1b1elp1b2* line 1 (*elp1b* #1) leaves. **c**, **d** Upon wounding a leaflet, the electrical signals were propagated through the rachillae basipetally (**c**) and acropetally (**d**) in *elp1b1elp1b2* line 8 (*elp1b* #8) leaves. Representative data are displayed (n = 10 for **a**; n = 6 for **b**; n = 16 for **c**; and n = 12 for **d**). Electrode positions are as illustrated in Fig. 2h (for **a** and **c**) and Supplementary Fig. 4a (for **b** and **d**).

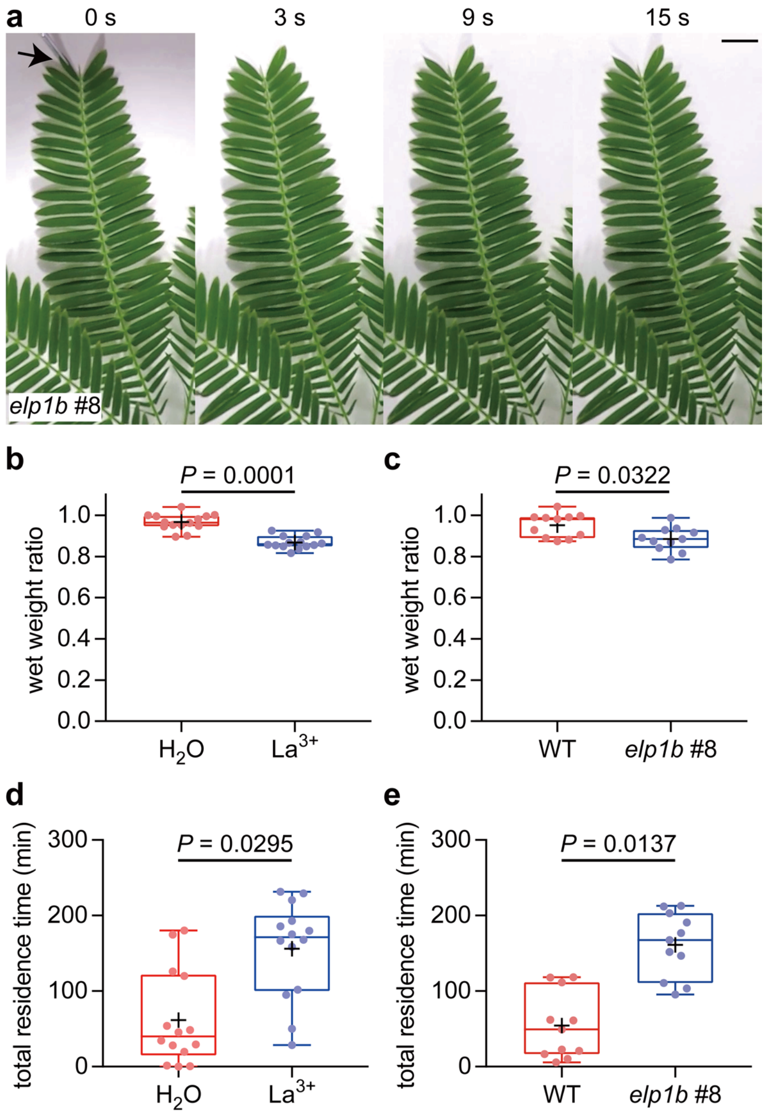

Supplementary Fig. 12 | Rapid leaf movement is a mechanical defense response against caterpillars. a Wounding (black arrow, 0 s) did not cause rapid leaflet movements in *elp1b1elp1b2* line 8 (*elp1b* #8) leaves. **b**, **c** Caterpillars fed more on the 50 mM La^{3+}-pretreated leaves (**b**) and *elp1b* #8 leaves (**c**) than on the control (WT) leaves. **d**, **e** Caterpillars stayed longer on the La^{3+}-treated leaves (**d**) and *elp1b* #8 leaves (**e**) than on the control (WT) leaves. n = 14 independent leaf pairs for **b** and **d**, and n = 11 independent leaf pairs for **c** and **e**. The boxes show the interquartile ranges, and the whiskers show the minimum and maximum values. The horizontal lines within the boxes and the plus signs indicate the medians and means, respectively. The dots represent individual data. Statistical analyses were performed using a two-tailed Wilcoxon matched-pairs signed rank test. Scale bars, 10 mm.

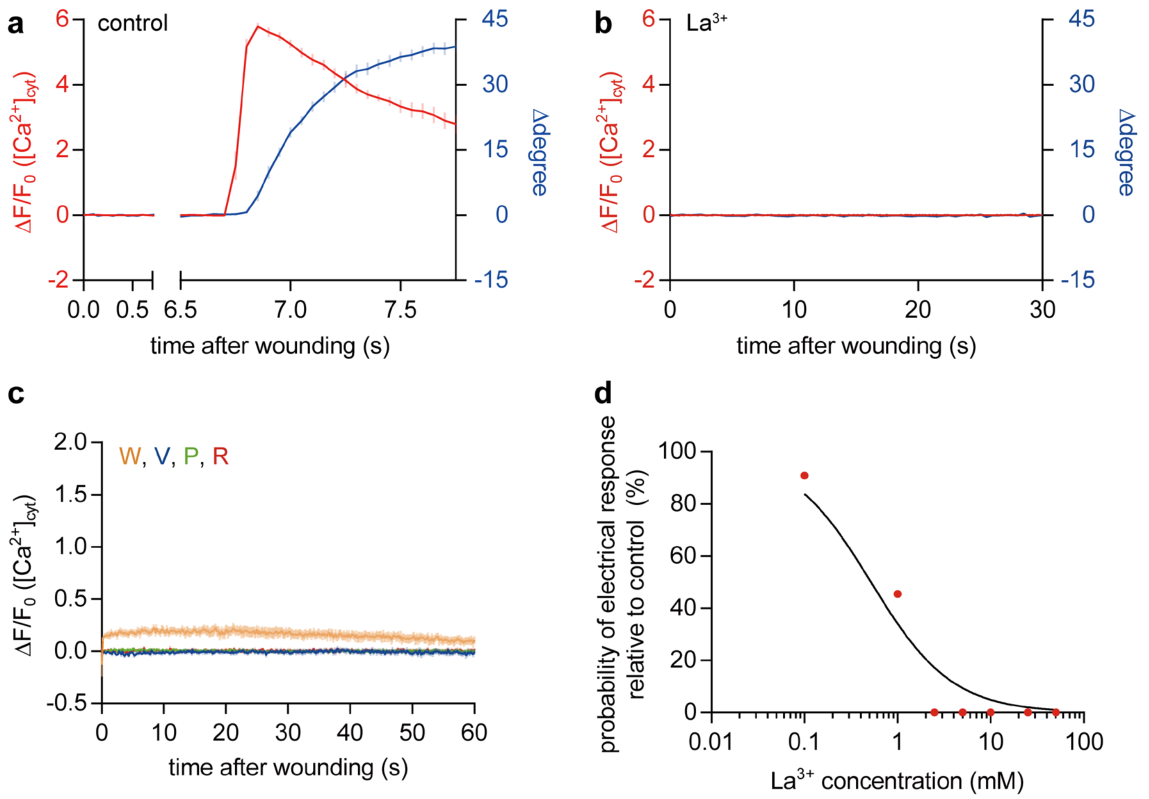

Supplementary Fig. 13 | La^{3+} solution with high K^{+} and Ca^{2+} concentrations inhibited long-distance Ca^{2+}/electrical signal propagation and leaflet movements. a, b $[Ca^{2+}]_{cyt}$ signatures at tertiary pulvini and leaflet angle changes in leaves pretreated with control (**a**, $n = 6$) and 50 mM La^{3+} solution (**b**, $n = 6$). **c** $[Ca^{2+}]_{cyt}$ changes at the wound site (W), leaflet vein (V), pulvinus (P), and rachilla (R) ($n = 8$ each). Mean ± SEM values are shown (**a**–**c**). For ROI positions, see Fig. 2b or Supplementary Fig. 3c. **d** Sigmoidal dose–response curve (black line). The probability of the electrical response at e1 (see Fig. 2h) relative to the control (red dots) was plotted against the La^{3+} concentration, for which IC_{50} was 0.52 mM.

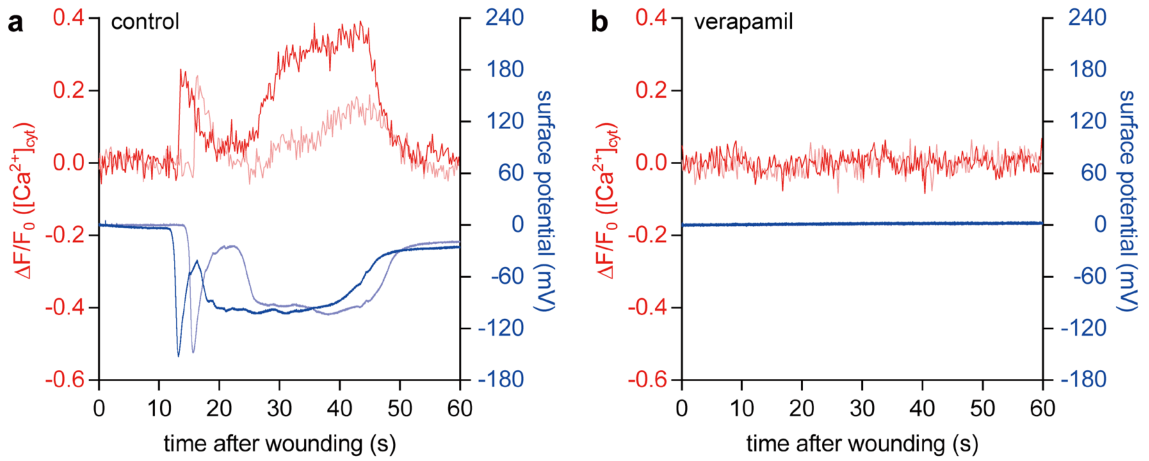

Supplementary Fig. 14 | Verapamil inhibits both $[Ca^{2+}]_{cyt}$ and electrical signals. a, **b** Wounding triggered $[Ca^{2+}]_{cyt}$ and electrical signals basipetally propagating in a rachilla in a control leaf (**a**) but not in a leaf treated with 2 mM verapamil (**b**). Typical data are displayed (n = 8 for **a**; n = 5 for **b**).

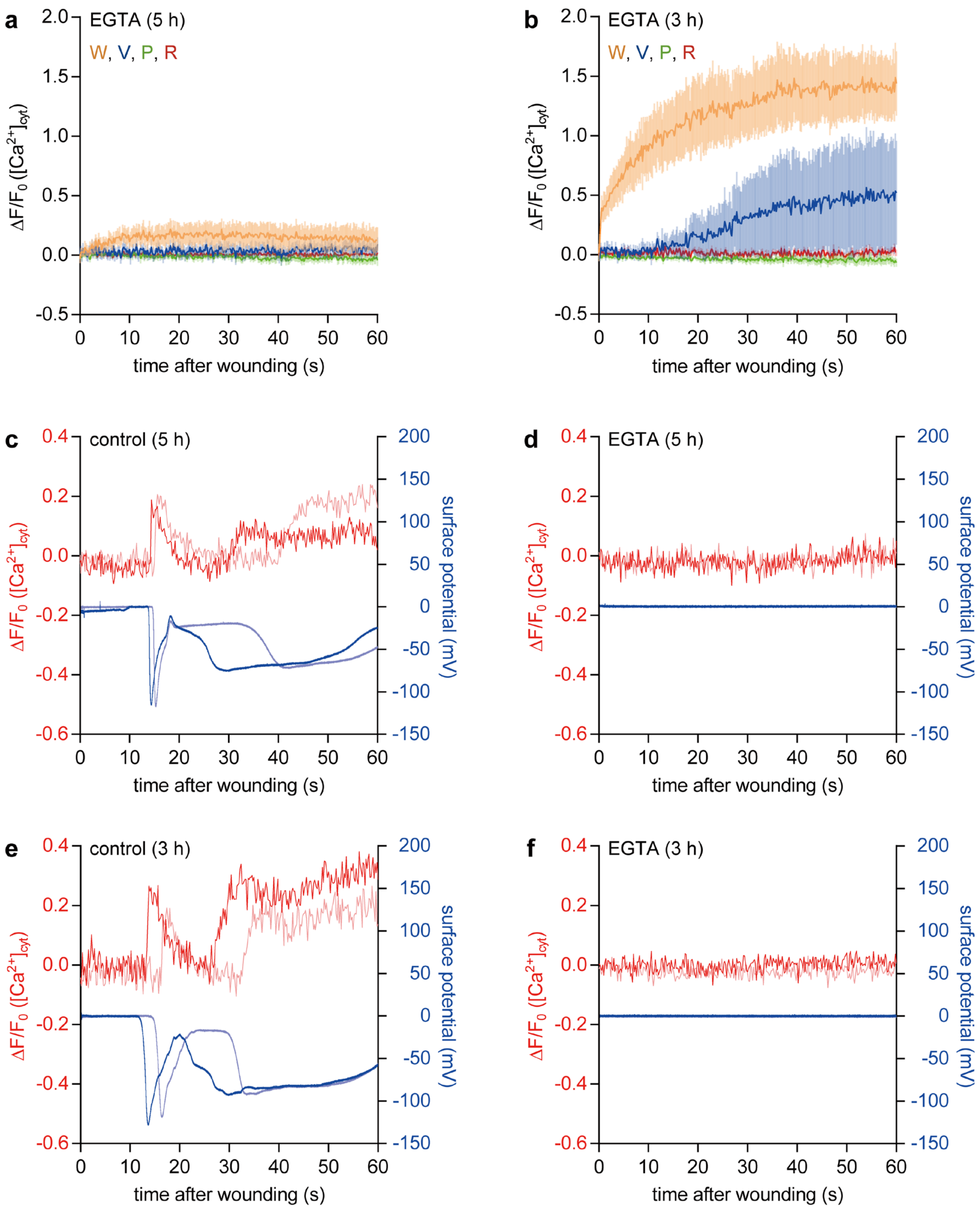

Supplementary Fig. 15 | The long-distance Ca^{2+}/electrical signal was inhibited by buffered EGTA solution. a, **b** $[Ca^{2+}]_{cyt}$ changes at the wound sites (W), leaflet veins (V), pulvini (P), and rachillae (R) in leaves treated with 50 mM EGTA for 5 (**a**, $n = 6$) or 3 h (**b**, $n = 6$). Mean ± SEM values are shown. For ROI positions, see Fig. 2b or Supplementary Fig. 3c. **c**, **d** Ca^{2+} and electrical signals were observed in the rachilla of a leaf treated with control solution for 5 h (**c**) but not in a leaf treated with 50 mM EGTA

solution for 5 h (**d**). The basipetal propagation of these signals was monitored. **e**, **f** Similar results were obtained when the treatment period was reduced from 5 to 3 h (**e**, control; **f**, EGTA). Representative data are displayed (**c** and **e**, n = 5; **d** and **f**, n = 8).

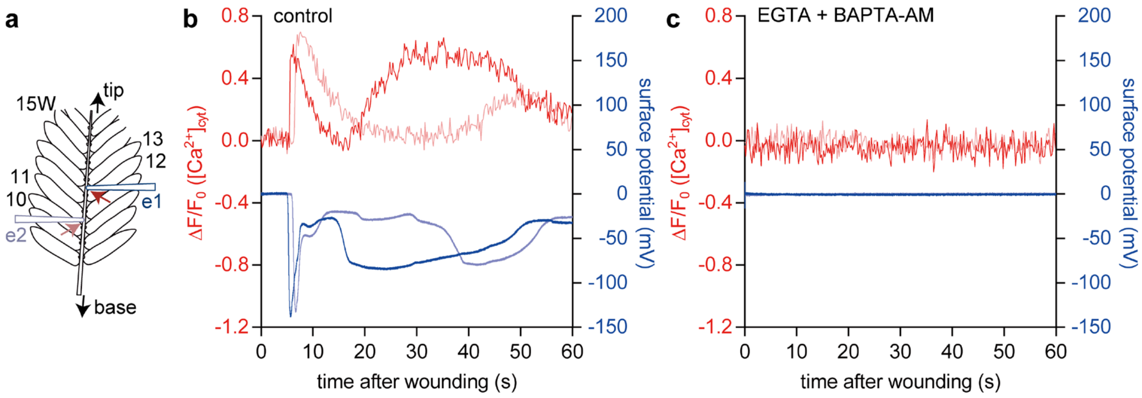

Supplementary Fig. 16 | Co-treatment of EGTA and BAPTA-AM to rachillae. a Electrodes (e1 and e2, blue rectangles) and ROIs (red arrows, 1 mm from the electrodes) were set on a rachilla for surface potential measurements and $[Ca^{2+}]_{cyt}$ analysis, respectively. A pair of leaflets was numbered from the base of a pinna. W, wounding. **b**, **c** Wounding triggered Ca^{2+} and electrical signals in the rachilla treated with control solution for 0.5 h (**b**), but not in the rachilla treated with 50 mM EGTA and ~1 mM BAPTA-AM for 0.5 h (**c**). Representative data are displayed (**b**, n = 5; **c**, n = 6).

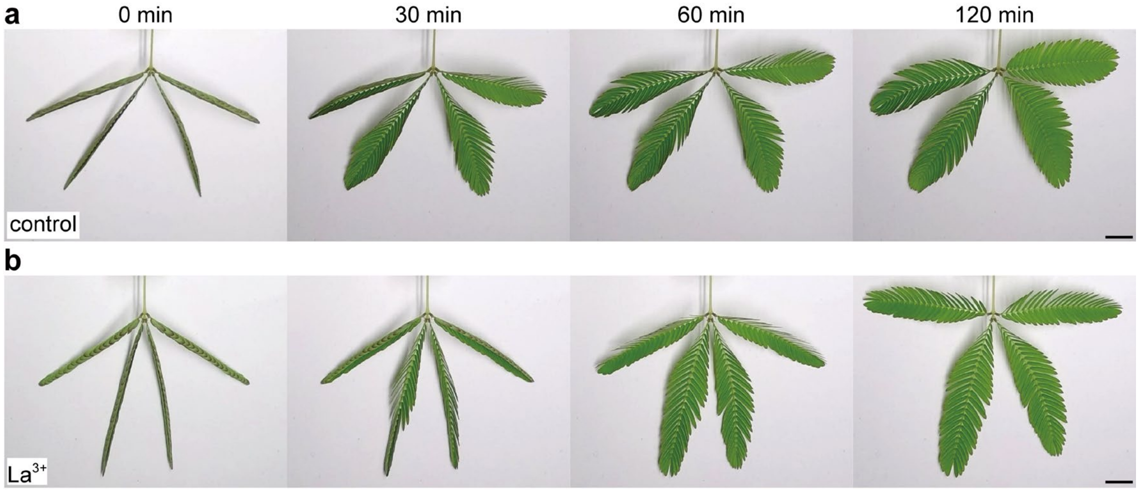

Supplementary Fig. 17 | La^{3+} does not inhibit the opening process of leaf movement. **a**, **b** Leaves were recovered in water (**a**) or 50 mM La^{3+} (**b**) and monitored for 2 h. Time was counted after the pharmacological treatment was started. Scale bars, 10 mm.

Supplementary Table 1 | Propagation rates of $[Ca^{2+}]_{cyt}$ and electrical signals in rachillae

Stimulus type	Measured signal	Direction of propagation	Velocity (mm/s)	*n*	*P*-value
Touch	$[Ca^{2+}]_{cyt}$	Basipetal	5.87 ± 0.75	6	0.6875
	Action potential		5.52 ± 0.43		
Wounding	$[Ca^{2+}]_{cyt}$ (1st peak)	Basipetal	4.13 ± 0.45	13	0.5879
	Action potential (1st peak)		4.27 ± 0.41		
	$[Ca^{2+}]_{cyt}$ (1st peak)	Acropetal	3.58 ± 0.34	11	0.8311
	Action potential (1st peak)		3.52 ± 0.29		

Statistical analyses were performed using a two-tailed Wilcoxon matched-pairs signed rank test.
