# Supplementary material for: Calcium-mediated rapid movements defend against herbivorous insects in Mimosa pudica
Source: Nat Commun. 2022 Nov 14;13:6412. doi: 10.1038/s41467-022-34106-x (PMC9663552; doi:10.1038/s41467-022-34106-x)
Supplement: Supplementary file 3 — Description of Additional Supplementary Files [file 41467_2022_34106_MOESM3_ESM.pdf]

Description of Additional Supplementary Files:

Supplementary Movie 1 | Touch-induced [Ca2+]cyt increases at tertiary pulvini and leaflet movements. The tip of a pinna was touched by tweezers (white arrow).

Supplementary Movie 2 | Wound-induced [Ca2+]cyt increases at tertiary pulvini and leaflet movements. A leaflet was wounded by scissors (white arrow).

Supplementary Movie 3 | Wound-induced [Ca2+]cyt increases at tertiary pulvini and leaflet movements in a leaf. A leaflet was wounded by scissors.

Supplementary Movie 4 | Wound-induced [Ca2+]cyt changes at tertiary pulvini and leaflet movements in a La3+ -treated leaf. A leaflet was wounded by scissors.

Supplementary Movie 5 | Wound-induced [Ca2+]cyt signal propagation in a leaf. The wounded site (white arrow) and [Ca2+]cyt signal propagation (yellow arrows) are indicated.

Supplementary Movie 6 | Wound-induced [Ca2+]cyt change in a La3+ - treated leaf. The wounded site is indicated by a white arrow.

Supplementary Movie 7 | Wound-induced [Ca2+]cyt change in a EGTA-treated leaf. The wounded site is indicated by a white arrow.

Supplementary Movie 8 | Wound-induced [Ca2+]cyt and electrical signals propagating basipetally through rachilla in a leaf. The wounded site (white arrow) and the [Ca2+]cyt signal propagation (yellow arrow) are indicated.

Supplementary Movie 9 | Wound-induced [Ca2+]cyt and electrical signals propagating basipetally through rachilla in a leaf. The wounded site (white arrow) and [Ca2+]cyt signal propagation (yellow arrow) are indicated. This movie is a control experiment for 50 mM La3+ treatment (Supplementary Movie 11).

Supplementary Movie 10 | Wound-induced [Ca2+]cyt and electrical signals propagating acropetally through rachilla in a leaf. The wounded site (white arrow) and [Ca2+]cyt signal

propagation (yellow arrow) are indicated. This movie is a control experiment for 50 mM La^{3+} treatment (Supplementary Movie 12).

Supplementary Movie 11 | Wound-induced $[Ca^{2+}]_{cyt}$ and electrical signals did not propagate basipetally through rachilla in a La^{3+} -treated leaf. The wounded site is indicated by a white arrow.

Supplementary Movie 12 | Wound-induced $[Ca^{2+}]_{cyt}$ and electrical signals did not propagate acropetally through rachilla in a La^{3+} -treated leaf. The wounded site is indicated by a white arrow.

Supplementary Movie 13 | Wound-induced $[Ca^{2+}]_{cyt}$ and electrical signals propagating basipetally through rachilla in a leaf. The wounded site (white arrow) and $[Ca^{2+}]_{cyt}$ signal propagation (yellow arrow) are indicated. This movie is a control experiment for 50 mM EGTA treatment (Supplementary Movie 15).

Supplementary Movie 14 | Wound-induced $[Ca^{2+}]_{cyt}$ and electrical signals propagating acropetally through rachilla in a leaf. The wounded site (white arrow) and $[Ca^{2+}]_{cyt}$ signal propagation (yellow arrow) are indicated. This movie is a control experiment for 50 mM EGTA treatment (Supplementary Movie 16).

Supplementary Movie 15 | Wound-induced $[Ca^{2+}]_{cyt}$ and electrical signals did not propagate basipetally through rachilla in an EGTA-treated leaf. The wounded site is indicated by a white arrow.

Supplementary Movie 16 | Wound-induced $[Ca^{2+}]_{cyt}$ and electrical signals did not propagate acropetally through rachilla in an EGTA-treated leaf. The wounded site is indicated by a white arrow.

Supplementary Movie 17 | Herbivory-induced $[Ca^{2+}]_{cyt}$ signals and leaflet movements. The feeding site is indicated by a white arrow. Note that the grasshopper is naturally fluorescent.

Supplementary Movie 18 | Herbivory-induced $[Ca^{2+}]_{cyt}$ signals and leaflet movements. The feeding site is indicated by a white arrow. Note that the grasshopper is naturally fluorescent.
