# Supplementary material for: Calcium-mediated rapid movements defend against herbivorous insects in Mimosa pudica
Source: Nat Commun. 2022 Nov 14;13:6412. doi: 10.1038/s41467-022-34106-x (PMC9663552; doi:10.1038/s41467-022-34106-x)
Supplement: Supplementary file 22 — Reporting Summary [file 41467_2022_34106_MOESM22_ESM.pdf]

nature portfolio

Corresponding author(s): Mitsuyasu Hasebe, Masatsugu Toyota

Last updated by author(s): Oct 11, 2022

Reporting Summary

Nature Portfolio wishes to improve the reproducibility of the work that we publish. This form provides structure for consistency and transparency in reporting. For further information on Nature Portfolio policies, see our Editorial Policies and the Editorial Policy Checklist.

Statistics

For all statistical analyses, confirm that the following items are present in the figure legend, table legend, main text, or Methods section.

n/a	Confirmed	
☐	☒	The exact sample size (n) for each experimental group/condition, given as a discrete number and unit of measurement
☐	☒	A statement on whether measurements were taken from distinct samples or whether the same sample was measured repeatedly
☐	☒	The statistical test(s) used AND whether they are one- or two-sided<br>*Only common tests should be described solely by name; describe more complex techniques in the Methods section.*
☐	☒	A description of all covariates tested
☒	☐	A description of any assumptions or corrections, such as tests of normality and adjustment for multiple comparisons
☐	☒	A full description of the statistical parameters including central tendency (e.g. means) or other basic estimates (e.g. regression coefficient) AND variation (e.g. standard deviation) or associated estimates of uncertainty (e.g. confidence intervals)
☐	☒	For null hypothesis testing, the test statistic (e.g. F, t, r) with confidence intervals, effect sizes, degrees of freedom and P value noted<br>*Give P values as exact values whenever suitable.*
☒	☐	For Bayesian analysis, information on the choice of priors and Markov chain Monte Carlo settings
☒	☐	For hierarchical and complex designs, identification of the appropriate level for tests and full reporting of outcomes
☒	☐	Estimates of effect sizes (e.g. Cohen's d, Pearson's r), indicating how they were calculated

Our web collection on statistics for biologists contains articles on many of the points above.

Software and code

Policy information about availability of computer code

Data collection	Image acquisition: NIS-Elements Advanced Research v4.30.02, ZEN pro (blue edition) v2.6.76.00000, Leica Application Suite v4.12.0<br>Electrophysiological measurement: Clampex 9.2
Data analysis	Statistical analysis: GraphPad Prism 7, Microsoft Excel v16.0.4266.1001<br>Image analysis: NIS-Elements Advanced Research v4.30.02, ZEN pro (blue edition) v2.6.76.00000<br>Electrophysiology data analysis: Clampfit 9.2, Microsoft Excel v16.0.4266.1001

For manuscripts utilizing custom algorithms or software that are central to the research but not yet described in published literature, software must be made available to editors and reviewers. We strongly encourage code deposition in a community repository (e.g. GitHub). See the Nature Portfolio guidelines for submitting code & software for further information.

Data

Policy information about availability of data

All manuscripts must include a data availability statement. This statement should provide the following information, where applicable:

- Accession codes, unique identifiers, or web links for publicly available datasets
- A description of any restrictions on data availability
- For clinical datasets or third party data, please ensure that the statement adheres to our policy

The data that support the findings of this study are available from the corresponding author upon reasonable request. Plasmid sequence data can be found in GenBank with the accession numbers pSB1 (AB027255) and pSB11 (AB027256). The GCaMP6f sequence is available at Addgene (#40755). ELP1B1 and ELP1B2 sequences are deposited in DNA Data Bank of Japan with the accession numbers ELP1B1 (LC731322) and ELP1B2 (LC731323). Source data are provided with this paper.

Field-specific reporting

Please select the one below that is the best fit for your research. If you are not sure, read the appropriate sections before making your selection.

- [x] Life sciences
- [ ] Behavioural & social sciences
- [ ] Ecological, evolutionary & environmental sciences

For a reference copy of the document with all sections, see nature.com/documents/nr-reporting-summary-flat.pdf

Life sciences study design

All studies must disclose on these points even when the disclosure is negative.

Sample size	No calculation was performed to pre-determine sample size. We conducted all experiments with sample sizes available at time when the experiments were performed.
Data exclusions	No data were excluded from the analyses.
Replication	Experiments were replicated as stated in the figure legends or methods.
Randomization	We randomly sampled in all experiments.
Blinding	Blinding was not conducted during data collection and analysis.

Reporting for specific materials, systems and methods

We require information from authors about some types of materials, experimental systems and methods used in many studies. Here, indicate whether each material, system or method listed is relevant to your study. If you are not sure if a list item applies to your research, read the appropriate section before selecting a response.

Materials & experimental systems

n/a	Involved in the study	
x		Antibodies
x		Eukaryotic cell lines
x		Palaeontology and archaeology
x		Animals and other organisms
x		Human research participants
x		Clinical data
x		Dual use research of concern

Methods

n/a	Involved in the study	
x		ChIP-seq
x		Flow cytometry
x		MRI-based neuroimaging
